# Modern proton therapy in prostate cancer: precision in practice

**DOI:** 10.3389/fonc.2026.1775107

**Published:** 2026-04-17

**Authors:** Irini Yacoub, Joshua Khalil, Shouyi Wei, Kristin Hsieh, Arpit M. Chhabra, Keyur J. Mehta, Caroline Oska, Lifei Zhu, Jehee Isabelle Choi, Charles B. Simone

**Affiliations:** 1 New York Proton Center, New York, NY, United States; 2 Department of Radiation Oncology, Memorial Sloan Kettering Cancer Center, New York, NY, United States; 3 Department of Biological Science, University of California San Diego, La Jolla, CA, United States; 4 Department of Radiation Oncology, Mount Sinai Medical Center, New York, NY, United States; 5 Department of Radiation Oncology, Montefiore Einstein Cancer Center, New York, NY, United States

**Keywords:** passive-scatter proton beam, pencil beam scanning, photon radiation, post-prostatectomy radiation, prostate, prostate and pelvic radiation, proton therapy

## Abstract

Proton therapy (PT) is an advanced form of radiation therapy that exploits the physical properties of the Bragg peak to deliver highly conformal dose distributions while minimizing radiation exposure to surrounding normal tissues. This precision is particularly relevant in prostate cancer, where critical organs at risk, including the rectum, bladder, bowel, and penile bulb, are in close proximity to the target. This review summarizes the evolution of PT techniques for prostate cancer, from early passively scattered proton therapy (PSPT) to contemporary intensity-modulated proton therapy (IMPT), and evaluates their dosimetric and clinical implications across multiple treatment settings. Dosimetric studies consistently demonstrate that PT, particularly IMPT, reduces integral dose and improves normal tissue sparing compared with photon-based modalities (XRT) such as intensity-modulated radiation therapy (IMRT) and volumetric modulated arc therapy (VMAT), especially in low- to intermediate-dose regions. These advantages are most pronounced for complex target volumes, including pelvic nodal irradiation and focal intraprostatic boosting. Clinical outcomes data for prostate only treatment indicate excellent disease control with low rates of high grade gastrointestinal and genitourinary toxicity. Emerging evidence suggests potential benefits of PT in reducing specific rectal symptoms and lowering the risk of secondary malignancies. For high-risk prostate cancer requiring pelvic irradiation, prospective and registry-based studies demonstrate favorable toxicity profiles with IMPT, supporting its use in extended treatment fields. In the postoperative setting, PT offers dosimetric improvements, though clinical benefits over XRT remain less clearly defined. Additionally, these advancements in technology have allowed for more precise hypofractionated treatment, including proton stereotactic body radiation therapy, without significant increases in genitourinary or gastrointestinal toxicity. Finally, ultra-high dose rate FLASH PT may further enhance the therapeutic ratio. Overall, modern PT represents a highly precise and evolving modality in prostate cancer management, with the potential to optimize oncologic outcomes while preserving long-term quality of life. Further randomized and cost effectiveness studies are needed to fully define its role relative to advanced photon techniques.

## Introduction

Proton therapy (PT) is a precise form of radiation therapy that minimizes radiation exposure of surrounding healthy tissues through modulation of multiple pristine Bragg peaks (1). Unlike conventional photon-based radiation therapy that generally has an exit dose, PT can release the bulk of its energy at a precise depth within the body, thereby limiting exit dose and better sparing surrounding normal tissues from excess radiation (1).

This precision is particularly beneficial in the treatment of prostate cancer, as it can reduce radiation exposure to nearby sensitive organs including the rectum, bowel, bladder and penile bulb. Consequently, the patients’ quality of life may be well-preserved or even improved after treatment by mitigating the risk of intestinal, urinary, and sexual side effects (2). Furthermore, the reduced integral dose associated with PT may decrease the risk of secondary malignancies (3). In one study analyzing over 450, 000 cancer patients treated with 3D-CRT, IMRT, or PT treatment with PT was associated with a substantially lower risk of developing a second cancer compared with IMRT and 3D-Conformal radiation (3D-CRT) (4). As a result, PT is a common indication for prostate cancer, to minimize the risk of rectal or bladder cancers (5). This review article evaluates the clinical utility of PT’s precision in the management of prostate cancer.

PT’s relative biological effectiveness (RBE) remains an active area of investigation; however, a pragmatic RBE value of 1.1 is commonly adopted in treatment planning systems to account for the modestly greater biological effectiveness of protons compared with photons (6). Linear energy transfer (LET), defined as the energy deposited per unit path length of tissue, is understood to increase toward the distal edge of the Bragg peak, paralleling the observed rise in RBE (7).

Proton RBE is not constant and generally increases with linear energy transfer (LET), so higher-LET regions (often near the distal edge) may elicit greater biological effect for the same physical dose. The LET–RBE relationship depends on tissue/endpoint radiosensitivity (commonly parameterized by photon α/β), so the RBE increase at a given LET can differ across tissues and clinical endpoints. RBE is also dose-dependent, including effects of dose per fraction, so fractionation can influence the apparent RBE even at comparable LET. Finally, factors such as oxygenation, cell type, repair kinetics, and model choice add uncertainty, while LET remains a primary practical correlate of spatial RBE variation. The proton doses reported in the cited studies and in this work are expressed as RBE-weighted dose in Gy(RBE), assuming a constant RBE of 1.1 applied to the physical dose.

## Materials and methods

A thorough literature review was conducted to identify the most relevant evidence regarding proton therapy in the management of prostate cancer. Searches were performed in major biomedical databases including PubMed, Embase, Scopus, Web of Science, and the Cochrane Central Register of Controlled Trials using combinations of keywords and subject headings related to prostate cancer, radiation therapy, and proton therapy techniques. Search terms included variations of “prostate”, “proton therapy”, “pencil beam scanning”, “passive-scatter proton beam”, “photon radiation”, “prostate and pelvic radiation” and “post-prostatectomy radiation.” Boolean operators (AND/OR) were used to combine search terms and optimize retrieval. Studies were screened based on relevance to clinical outcomes, dosimetric comparisons, and toxicity endpoints associated with proton therapy. Review articles, case reports, non-clinical studies, and studies lacking relevant clinical endpoints were excluded. Eligible studies included comparative analyses, prospective or retrospective clinical studies, and dosimetric planning studies evaluating proton therapy in prostate cancer. The selected literature formed the basis for the present review of modern proton therapy techniques and their clinical implications in prostate cancer management.

### Double scattering proton radiation

Early generations of PT, including for the treatment for prostate cancer, commonly utilized double-scattering also referred to as PSPT. With PSPT, a focused, monoenergetic proton beam generated by a synchrotron or cyclotron first interacts with a high-Z (e.g. tantalum, tungsten, lead) scatterer resulting in large-angle Coulomb scattering. The beam is then further broadened and spatially homogenized by a subsequent low-Z (e.g. aluminum, carbon/graphite, plastics like PMMA) scatterer, producing a uniform lateral fluence distribution.

The scattered beam is subsequently shaped laterally by a patient-specific aperture that conforms to the target outline in the beam’s eye view, while its depth is modulated by a range compensator to align the distal fall-off of the Bragg peak with the target’s distal surface. In double-scattering treatment for prostate cancer, two opposed lateral fields are typically employed to achieve a uniform dose coverage to the prostate while minimizing exposure to adjacent organs at risk (OARs), such as the rectum and bladder. The customized aperture openings play a key role in limiting the dose to these critical structures (8).

Dosimetric analyses have compared PSPT to IMRT. In one comparison by Trofimov et al., 10 patients were planned with both PSPT and IMRT to a total dose of 79.2 Gy(RBE) to the prostate gland. Compared to the PT plans, the IMRT plans irradiated substantially greater volume of tissue in the low dose range (defined as below 30 Gy(RBE)) and less volume in the 50–75 Gy(RBE) dose range. While the IMRT plans were more conformal to the target, they had hot spots of up to 115% of the prescription dose, whereas the PT provided better dose-homogeneity within the target. Additionally, PT delivered excess dose to the areas immediately adjacent to the target due to a broader penumbra and the compensator smear effect, whereas IMRT delivered higher doses to healthy tissue elsewhere. PT also significantly reduced mean doses by 26% to the rectum, and by 20% to the bladder, compared to IMRT (9).

Yeo and colleagues performed a similar comparison, although their study included a focal tumor boost to 90 Gy(RBE). While IMRT plans were demonstrated as more conformal, PT plans had superior homogeneity. Additionally, while both the IMRT and PT plans resulted in similar rectal volumes receiving doses >65 Gy(RBE), the volumes receiving doses ≤65 Gy(RBE) were decreased for PT, resulting in an integral dose that was 45.6% lower to the rectum and 40.9% lower to the rectal wall. Normal tissues outside the planning target volume also received an integral dose that was 36% less with the PT plans compared to that of IMRT plans. However, PT plans had significantly higher doses delivered to the left femoral heads that was attributed to the left lateral proton beam boosting employed to deliver the focal tumor boost with PT (10).

Although there appears to be some advantages to PSPT, there are several disadvantages. Firstly, these systems are sensitive to variations in the radiation beam position. Additionally, the use of the scattering system and aperture can produce secondary neutrons, which may increase whole-body radiation exposure to the patient. There are also additional time and cost efforts needed to manufacture and QA patient-specific apertures and compensators, limiting the overall efficiency of treatment. Furthermore, due to the characteristics of the spread-out Bragg peak, the high-dose region needs to be retracted to avoid the Bragg peak depositing the dose in adjacent critical normal structure, making the use of this system less optimal for lesions that conform closely around critical OARs (1).

### Pencil beam scanning proton radiation

Pencil beam scanning (PBS) is the dominant delivery technique in modern IMPT. In PBS, a narrow proton pencil beam is transported through the beamline and laterally positioned by two orthogonal scanning magnets located in (or immediately upstream of) the treatment nozzle. The dose is delivered by “painting” the target in the transverse plane using either spot scanning or raster scanning. In spot scanning (step-and-shoot), the magnets position the beam at a discrete spot coordinate; the beam is then enabled to deliver the prescribed spot weight (monitor units/protons) and subsequently turned off or gated to near-zero while the magnets transition to the next spot. In raster scanning (continuous line scanning), the beam is swept continuously along a line (often in a serpentine pattern), and the delivered fluence is controlled by synchronizing scan speed and/or beam current/extraction; the beam may remain on during the sweep, with brief beam interruptions as needed for delivery transitions and safety monitoring.

Depth modulation is achieved by changing proton energy between layers. In cyclotron-based PBS, energy changes are commonly produced using a variable energy degrader (with an energy selection system) followed by beam transport and refocusing to restore beam quality. In synchrotron-based PBS, the desired energy is selected within the accelerator cycle and protons are extracted at that energy (i.e., without requiring an external degrader for each layer). By combining transverse scanning (spot or raster) with layer-wise energy changes, PBS enables intensity- and energy-modulated PT with improved dose conformity and normal-tissue sparing compared with passive scattering (6).

In prostate PBS treatments, lateral opposing beams remain the standard configuration, providing a stable and reproducible setup. Single-field optimization (SFO) techniques are often delivered to enhance plan robustness while maintaining adequate sparing of OARs. For cases involving extended targets, such as prostate and pelvic nodes, multi-field optimization or intensity-modulated proton therapy (IMPT) is typically employed to achieve improved dose conformity and enhanced sparing of critical structures, particularly the bowel and rectum.

### Dosimetric benefits of proton therapy

Whether the previously mentioned advanced proton delivery system translates to improved dosimetry compared to XRT and PSPT has been analyzed in dosimetric studies. In one comparison by Takagi et al. (11), 30 patients were planned to a dose of at least 73 Gy(RBE) using PBS plans, specifically the line scanning (LS) proton plan, as well as PSPT plans, and photon-based VMAT plans. LS is a form of PBS where the beam is scanned continuously along straight lines, as opposed to discrete points (12). The authors found that the LS plans consistently met all strict dose constraints (e.g., rectal V65 < 17%, V40 < 35%; bladder V65 < 25%, V40 < 50%), whereas some PSPT and VMAT plans failed to satisfy the rectal and bladder limits. Across most dose–volume metrics, LS achieved lower irradiated volumes of the rectum and bladder, particularly in low- to intermediate-dose regions (V10–V70). While all three maintained adequate target coverage, PS and VMAT delivered higher integral doses to surrounding tissues. Among the three modalities, LS provided the best overall dosimetric balance, achieving high conformity and steep dose falloff around the prostate (11).

In another dosimetric analysis by Ong et al. (13) comparing PBS protons and VMAT, 10 patients with high-risk prostate cancer had two RT plans with a boost to the dominant intraprostatic lesion (DIL) using IMPT and VMAT. Across all patients, IMPT achieved a higher mean dose (Dmean) coverage for the DIL and a higher tumor control probability compared to those of VMAT. Additionally, IMPT plans resulted in lower intermediate- to high-dose exposure to the rectum and bladder. The rectum and bladder also showed reduced mean doses (Dmean), with average decreases of 15.9% and 14.9%, respectively, compared with VMAT (13).

Finally, a similar dosimetric comparison for patients receiving RT to the pelvic nodes for unfavorable or high risk prostate cancer demonstrated not only lower mean doses to the bladder, rectum, large bowel, and small bowel with IMPT, but the percentages of rectum receiving ≤ 47.5 Gy(RBE), large bowel receiving ≤ 27.5 Gy(RBE), small bowel receiving ≤ 30 Gy(RBE), and bladder receiving ≤ 37.5 Gy(RBE) were all significantly less with IMPT versus VMAT, largely owing to a reduction in the low-dose “bath” associated with VMAT (14). Overall, these dosimetric analyses demonstrate excellent tumor volume coverage with superior normal tissue sparing with IMPT compared to both VMAT and PSPT.

### Prostate only RT

Due to the long life expectancy of men with localized prostate cancer, efforts to minimize long term toxicities secondary to treatments are important and necessary. The use of PT in this setting has been explored as a way of decreasing urinary, bowel, and sexual toxicity (**Table 1**).

**Table 1:** Prostate-only radiation therapy studies.

Author	Number of patients treated	Cancer outcomes	Toxicity outcomes
Bryant et al.	1327	5-yr FFBP: 99% low, 94% intermediate, 74% high risk	Late ≥G3: GI 0.6%, GU 2.9%
Efstathiou et al.	450; PBT 221, IMRT 216	5-yr PFS 93.4% PT vs 93.7% IMRT (p=0.706)	No difference for bowel score at 24 months or earlier/later time points. No differences were observed in other domains (urinary, sexual, hormonal) at any timepoint
Hoppe et al.	1243 PT; 204 IMRT	Comparable disease control	PT showed less rectal urgency & bowel frequency; similar overall QOL
Corrao et al.	160 studies pooled	5-yr biochemical RFS 95% PT vs 91% photon	Acute ≥G2 GI 2% PT vs 7% XRT
Bao et al.	334 unmatched cohort; 177 PBT, 157 IMRT. 176 matched (88 PNT, 88 IMRT)	9-yr biochemical control 91% PT vs 85% IMRT for matched cohort	Secondary cancers 0.6% PT vs 4.5% IMRT

In a large study by Bryant et al. of 1327 men treated with 78 Gy(RBE) or higher to the prostate, at a median follow-up of 5.5 years, the authors reported late 5-year grade ≥3 gastrointestinal (GI) and genitourinary (GU) adverse event rates of 0.6% and 2.9%, respectively. Five-year freedom from biochemical progression was 99%, 94% and 74% for low, intermediate, and high-risk patients, respectively (15). However, it is unclear if the toxicity rate with PT is significantly better than that with XRT in these patients given lack of comparison.

The PARTIQoL trial randomized patients with low to intermediate risk prostate cancer to either PBT (PSPT and PBS) or IMRT, and followed them longitudinally to asses patient reported outcomes of bowel, urinary, and sexual function for 60 months after treatment. The primary endpoint was changes in baseline bowel quality of life (QOL) at 24 months. This trial did not demonstrate a statistically significant difference in patient reported bowel, urinary and sexual QOL outcomes, at any time point, between the two treatment modalities at 5-years. Additionally, there was no difference in progression-free survival (PFS) (93.4% vs 93.7%, p=0.706) at 60 months, between the two arms (16). However, 51% of the proton cohort in this study received PSPT, which may have diluted the benefit of PT now achievable with PBS. In another prospective study by Hoppe et al., patient-reported quality-of-life outcomes were compared between (PT and IMRT for localized prostate cancer using the EPIC questionnaire. The Expanded Prostate Cancer Index Composite (EPIC-26) is a validated survey tool composed of five domains: urinary incontinence (UI; 4 items), urinary irritative/obstructive symptoms (UO; 4 items), bowel function (6 items), sexual function (6 items), and hormonal function (5 items). Each domain is scored on a scale from 0 to 100, where higher scores indicate better function (no symptoms) and lower scores reflect more severe or significant problems within that domain. The study included 1, 243 men treated with PT at a single institution to doses of 76–82 Gy(RBE) and 204 men treated with IMRT from the multicenter PROSTQA cohort to doses of 75.6–79.4 Gy(RBE), with EPIC surveys collected at baseline and at multiple post-treatment time points. Overall bowel, urinary, and sexual summary scores were similar between groups at 6 months, 1 and 2 years; however, IMRT patients reported significantly higher rates of rectal urgency and increased bowel frequency at 6-months, 1 and 2-years, and fewer PBT patients with minimally detectable 6-month bowel scores from baseline (P = .002). These findings suggest that while global quality-of-life outcomes are comparable, PT may reduce specific rectal symptoms that meaningfully affect long-term patient quality of life (17).

A large meta-analysis and systematic review of 160 studies comparing PT and XRT analyzed GU and GI toxicity outcomes. The authors reported significantly lower rates of grade 2 or higher acute GI adverse events with proton RT compared to XRT (2% versus 7%), and improved 5-year biochemical relapse-free survival (95% vs 91%) were observed in the PT arm compared to XRT (18). Finally, in a case-matched analysis of 334 patients treated with PT versus XRT, the authors reported comparable biochemical failure-free survival at a median follow-up of 9 years (85% for IMRT vs 91% for PT; *p* = .39). More notably, the proton cohort demonstrated a significantly lower incidence of secondary cancers, defined as non-prostate malignancies arising within the irradiated field after a latency of at least 5 years, compared with the photon cohort (0.6% vs 4.5%) (19), similar to other population-based analyses showing significant reductions in secondary cancer risks following radiation therapy for prostate cancer with PT relative to XRT (4).

Overall, while both PT and IMRT may provide effective disease control for localized prostate cancer, current evidence suggests that PT may offer certain long-term advantages, particularly in reducing treatment-related side effects and the risk of secondary malignancies.

### Radiation of the pelvis

Clinical target volumes for high-risk prostate cancers typically encompass both the prostate and the pelvic nodes. Due to their location, coverage of the common and internal/external iliac vessels leads to a large volume of healthy tissues exposed to higher integral dose from IMRT. One potential benefit of IMPT is lower integral dose to the bowel and bladder despite large target volumes (**Table 2**).

**Table 2:** Pelvic nodal/high-risk prostate radiation studies.

Author	Number of patients treated	Cancer outcomes	Toxicity outcomes
Choo et al. (acute)	55	Not reported	During RT: GI G1 62%, G2 2%; GU G1 65%, G2 35%; no G3+ 3 months after RT: GI G1 9%, G2 0%; GU G1 80%, G2 14%
Choo et al. (5-yr)	56	5-yr recurrence-free rate 90%; DFS 89%	Late GI ≥2 16%, ≥3 4%; Late GU ≥2 41%, ≥3 0%
Hasan et al.	605	5-yr MFS 93%; OS 96%	Acute GU G2 21.8%, G3 0%; Late GU G2 5.8%, G3 1.7%; Late GI G2 5%; 2-year ED G2 48%; G3 8.4%

Choo et al. evaluated acute GU and GI toxicities of IMPT targeting prostate, seminal vesicle (SV), and pelvic lymph nodes. Genitourinary (GU) symptoms were assessed using the National Cancer Institute Common Terminology Criteria for Adverse Events (CTCAE), EPIC-26, the Patient-Reported Outcomes version of the CTCAE, and the AUA Symptom Index. Gastrointestinal (GI) symptoms were evaluated with the CTCAE. GI toxicity was measured across seven categories: diarrhea, fecal incontinence, proctitis, rectal hemorrhage, rectal stenosis, rectal ulcer, and small-intestinal obstruction. GU toxicity was assessed in nine categories, including cystitis, urinary frequency and urgency, obstruction and retention, urinary pain, bladder spasm, incontinence, and hematuria. Fifty-five patients received 6750 cGy(RBE) to the prostate and 4500 cGy(RBE) to the pelvic nodes. 62% and 2%, respectively, experienced acute grade 1 and 2 GI toxicity during radiation, which declined to 9% and 0% at 3-months after radiation. The 3 most common GI toxicities were diarrhea, proctitis, and rectal hemorrhage. Additionally, 65%, and 35% experienced grade 1 and 2 GU toxicity during radiation, and 80% and 14%, experienced grade 1 and 2 GU toxicity at 3 months after RT, respectively. The three most common grade 2 GU adverse events were urinary frequency, urgency, and obstructive symptoms. No patient reported acute grade 3 or higher GU or GI adverse events (20). Five-year follow-up of this prospective study reported late grade ≥2 and ≥3 GI adverse events of 16% and 4%, respectively, and late grade ≥2 and ≥3 GU adverse events rates of 41% and 0%, respectively, at 5-years, Recurrence free rate and disease free survival at 5-years were 90% and 89%, respectively (21).

In a larger analysis using the Proton Collaborative Group prospective registry by Hasan et al. (22), outcomes of 605 men with HR prostate cancer treated with PT were evaluated. Median dose was 79.2 Gy(RBE) in 44 fractions, and 58% of patients received pelvic RT. At a median follow-up of 22 months, 5-year metastasis-free survival and OS were 93% and 96%, respectively. Acute grade 2 and 3 GU side effects, defined as occurring within 3 months of RT, were reported in 21.8% and 0%, of patients respectively, whereas late grade 2 and 3 GU AE (occurring beyond 3 months after RT) were reported in 5.8% and 1.7% of patients, respectively. Late Grade 2 and 3 GI AE were reported in 5%, and 0%, respectively. No acute GI events were reported in this paper. Additionally, they reported a prevalence rate of 48.4% and 8.4% for grade 2 and 3 erectile dysfunction at 2 years, respectively (22).

Overall, PT, particularly IMPT, can reduce the integral dose to the bowel and bladder, particularly in extensive treatment fields, such as those including the pelvic nodes as is needed for high-risk prostate cancers (23). As demonstrated above, clinical data show that IMPT achieves excellent target coverage with low rates of acute and late GI and GU toxicities, with most adverse effects being mild to moderate (20) (21). Large registry analyses confirm these favorable toxicity profiles, supporting PT as a safe and effective option for patients with HR prostate cancer with pelvic nodal irradiation (22).

### Postoperative pelvic radiation

Literature on the use of PT in the post-prostatectomy setting is limited, but the few available studies have generally shown improved dosimetric results. A 2010 report by Doyle et al. compared photon-based IMRT and VMAT to proton RT plans. The authors found improvement in rectal doses with proton plans, as well as bladder doses at V30 and V17.1Gy(RBE) (24). However, it is unclear if these dosimetric improvements necessarily translate to a meaningful clinical benefit. Two studies found in the literature report on the use of PT in this setting (**Table 3**).

**Table 3:** Postoperative/salvage setting studies.

Author	Number of patients treated	Cancer outcomes	Toxicity outcomes
Kharod et al.	102	5-year FFBP, DMFS, and OS: 57%, 97%, and 93%, respectively	Acute ≥G3 GU 1%; Late ≥G3 GU 7%; no ≥G3 GI Acute G2 GI 5%; late G2 GI 2%.
Santos et al.	307	Not reported	Five-year grade ≥2 GU and grade ≥1 GI toxicity free survival (TFS) 61.1% and 73.7% for IMRT and 70.7% and 75.3% for PBT, respectively; Five-year grade ≥3 GU and GI TFS was >95% for both groups (all p≥0.05).

Kharod et al. performed a retrospective analysis of 102 men treated between 2006 and 2017 in the Proton Collaborative Group registry. All participants received double-scattered PT directed to the prostate bed, with or without pelvic nodal coverage, at a total dose range of approximately 66–78 Gy(RBE). At a median follow-up of 5.5 years, 5-year biochemical recurrence free survival and distant metastases free survival were 72% and 91% for adjuvant, and 57% and 97% for salvage patients. Reported toxicities were generally minimal. No patient experienced grade 4 or 5 acute (defined as during or within 6 months of RT) or late (>6 months after RT) adverse events. A single patient (1%) developed acute grade 3 GU toxicity, and 7% experienced late grade 3 GU complications, including dysuria (1%), urinary frequency (1%), incontinence (2%), retention (1%), or hematuria (2%), all of which resolved. There were no grade 3 or higher GI events. Grade 2 GI symptoms occurred acutely in 5% of patients, most commonly diarrhea (3%), abdominal cramping (1%), or proctitis (1%). Only two (2%) patients developed late grade 2 GI issues, specifically rectal bleeding that subsided over time (25).

Conversely, Santos et al. conducted a retrospective, case-matched comparison of post-prostatectomy PT versus IMRT, evaluating acute and late GU and GI toxicities as primary endpoints. For both cohorts, the median prescription dose was 70.2 Gy(RBE). PT plans were significantly better at minimizing low-range dose (V10-V40) to bladder and rectal structures and high-range dose (V50–65) to the anterior rectal wall. However, this did not translate to a significant clinical difference. Median follow-up was 48.6 and 46.1 months for IMRT and PBT, respectively. Most matched patients experienced only mild acute (defined as within 90 days of RT) gastrointestinal (GI) toxicity, with grade 1 events reported in 39.6% of IMRT and 27.1% of PT patients. Aside from a slightly higher rate of rectal pain with IMRT (10.7% vs 2.9%, *p* = 0.049), there were no significant differences in other acute GI toxicities, and no grade ≥3 acute events occurred in either group. Late grade ≥2 genitourinary (GU) toxicity–free survival at 1, 2, and 5 years was similar between IMRT (72.5%, 66.8%, and 61.1%) and PT (72.5%, 70.7%, and 70.7%) (*p* = 0.20), with grade ≥3 toxicity–free survival exceeding 95% for both groups. Late grade 3–4 GU toxicities occurred only in the IMRT cohort and were rare. Concurrent androgen deprivation therapy and diabetes were associated with higher odds of late GU toxicity, whereas treatment modality itself was not significantly associated with risk (26).

These conflicting results highlight the need for prospective randomized studies comparing the two treatment modalities of PT and IMRT. It is noteworthy to mention that in the post-prostatectomy setting, it is often difficult to distinguish the side effects attributable to radiotherapy versus those resulting from surgery, with post-surgical side effects being a common factor that may potentially obscure any observable improvement in GU- or GI-related side effects between the proton and photon treatments.

### Future directions

The next phase of PT in prostate cancer will be informed by both emerging comparative data and continued technological innovation. The COMPPARE trial, a prospective study comparing patient-reported outcomes and toxicity between PT and IMRT, has completed accrual and is pending publication (NCT03561220). Its results are expected to provide critical prospective evidence regarding the relative clinical benefits of modern PT.

In parallel, hypofractionated and stereotactic body radiation therapy (SBRT) with protons is gaining clinical support, including initial randomized data from the PCG GU 002 trial demonstrating favorable quality of life outcomes, and low toxicity with hypofractionated PT, compared with conventional fractionation (27, 28). A recent national consensus survey has further characterized current practice patterns for proton SBRT, and outlined recommendations to help standardize delivery and expand access across proton centers (29). Looking ahead, ultra-high dose rate FLASH PT holds promise for reducing normal tissue toxicity based on robust preclinical evidence, with the first human trial recently reported (30), and early feasibility data now available specifically for prostate cancer using Bragg-peak based FLASH SBRT approaches (31).

Beyond physical dose optimization, biologically guided planning strategies are increasingly being explored. LET optimization extends prostate proton planning beyond physical dose by explicitly steering dose-averaged LET, because LET tends to rise near distal falloffs and may elevate biological effect in nearby OARs. Methods proposed in the proton literature include multi-criteria LET-guided optimization to increase LET in the target while reducing LET in normal tissues (32) as well as integrating LET (or LET×dose surrogates) directly into IMPT objective functions (33). In prostate, this motivation is strengthened by clinical/planning evidence linking combined high dose + high LET metrics to rectal toxicity risk (e.g., dose-LET volume histogram style predictors of rectal bleeding) (34), and by more recent robust optimization framework that explicitly minimize high-dose/high-LET overlap in the rectum and bladder while preserving target dose robustness (35).

Building further on biologic optimization, multi-ion approaches combining proton treatment with a heavier ion such as helium or carbon, are motivated by the ability to mix complementary physical and LET characteristics across ions to shape biological effect, e.g., using protons for robust uniform coverage while leveraging a heavier ion component for a tighter, potentially higher-LET boost to selected intraprostatic subvolumes (with explicit constraints to avoid placing high-LET regions in rectum/urethra). Reviews describe this “multi-ion” concept and its potential to tailor LET distributions within tumors (36), and recent treatment-planning work has proposed biological dose optimization schemes that automatically adjust the relative contributions of protons and carbon ions within a combined plan (37).

Together, prospective comparative trials, adaptive and hypofractionated delivery strategies, FLASH irradiation, LET-guided optimization, and multi-ion biological planning represent converging efforts to refine both the physical and biologic precision of PT. As these approaches mature and clinical validation expands, they hold promise for further improving oncologic outcomes while enhancing long-term tolerability for patients with prostate cancer.

## Conclusion

Modern PT represents a significant advancement in the precision and personalization of radiotherapy for prostate cancer. Through innovations such as IMPT, clinicians can achieve superior target conformity and normal tissue sparing compared to conventional photon-based techniques and older PSPT. These dosimetric advantages translate into reduced integral dose to OARs, including the rectum, bladder, and bowel, potentially minimizing both acute and late GU and GI adverse events. Evidence from clinical and registry studies demonstrates excellent disease control outcomes and favorable side effect profiles with PT, particularly for HR patients requiring pelvic nodal irradiation. Although the benefits of PT are most apparent in complex or extensive treatment volumes, studies are needed to confirm its long-term clinical advantages and cost-effectiveness compared with advanced photon modalities. In the postoperative setting, early data suggest promising dosimetric improvements, although definitive evidence of clinical benefit remains limited. As technology continues to evolve, integrating robust motion management, adaptive planning, and biologically optimized dose modeling will further refine proton therapy’s role in prostate cancer care. Ultimately, the precision of modern proton therapy offers an opportunity to balance oncologic control with the preservation of quality of life in a disease where treatment precision and survivorship outcomes are paramount.
